# Typhoidal Insanity in Childhood

**Published:** 1905-04-15

**Authors:** 


### TYPHOIDAL INSANITY IN CHILDHOOD.

Dr. Edsall[1] has collected some valuable data as to the varieties and gravity of the insanities associated with typhoid fever. He states that authorities are much at variance as regards the prognosis in the case of these psychoses, Dr. Osler speaking most hopefully about them, while Dr. Clouston is of opinion that they rarely recover. The explanation of this anomaly offered by the author is that alienists are likely to see the most confirmed cases. Eighty-three cases of such insanities in childhood have been collected, the varieties being simply grouped as follows:—Mania, 36; dementia, 26; melancholia, 6; simple delusions of convalescence, 14; chronic paranoia, 1. Dr. Edsall considers that the melancholias are much under-estimated, for most of the maniacal cases show a strong undercurrent of melancholy, though an occasional outburst of maniacal excitement leads to their inclusion in the maniacal class.

As regards prognosis, all the cases showing the simple delirium and delusions of convalescence recovered completely. Of the remaining 69 there recovered 43, or 62·3 per cent.; there died 3, or 4·34 per cent.; remained insane, 23 or 33·3 per cent.

The relative gravity of the different types is shown in the following table:—

| | Recovered. | Died. | Remained insane. |
|---|---|---|---|
| Of 36 manias ............ | 29 | 2·5 | 5 |
| Of 6 melancholias ... | 5 | 0 | 1 |
| Of 26 dementias ......... | 9 | 1 | 16 |

This leaves the proportion of cures as follows:—Mania, 80·5 per cent.; melancholia, 83·4 per cent.; dementia, 34·6 per cent.

Dementia, therefore, is by far the gravest complicating psychosis, and, if the figures might be implicitly believed, a little-reckoned risk of typhoid fever would seem to command most alarming proportions. But the author hastens to disavow acceptance of these figures as they stand, for he justly observes that the severe cases are inherently the most likely to be placed on record, and admits that a considerable number of instances in the series were reported by alienists.

Strangely enough no success attended an effort to trace the influence of neuropathic inheritance in these cases, but the accessible details made the investigation somewhat unreliable. Touching the period of the illness most likely to be attended by mental disturbance, it appeared in a large majority—54 out of 65—during convalescence.

Here, again, the author inclines to discredit the record on the score of the difficulty which, in a child, attends the distinction between the delirium of fever and the early stages of a more serious psychosis. There appears to be little or no selection of subjects according to age limits. In a series of 67 cases, 14 were children in the first hemi-decade, the second supplied 26, and the third 27.

In the matter of treatment Dr. Edsall says that all the melancholic cases showed marked evidence of nutritional failure (which supplies an indication for particular attention to as generous a régime as circumstances allow), and insists that this psychosis is often aggravated by homesickness. He has remarked a rapid improvement upon the return of such melancholic patients from public institutions to their homes.

[1] Amer. Jour. of Med. Sci., Feb. 1905.

